# Association of the cumulative triglyceride-glucose index with major adverse cardiovascular events in patients with type 2 diabetes

**DOI:** 10.1186/s12933-022-01599-1

**Published:** 2022-08-23

**Authors:** Shi Tai, Liyao Fu, Ningjie Zhang, Rukai Yang, Yuying Zhou, Zhenhua Xing, Yongjun Wang, Shenghua Zhou

**Affiliations:** 1 Department of Cardiovascular Medicine, The Second Xiangya Hospital of Central South University, Changsha, China; 2 Department of Blood Transfusion, The Second Xiangya Hospital of Central South University, No. 139, Middle Renmin Road, Changsha, 410011 Hunan People’s Republic of China; 3 Department of Cardiology, The Xiangtan Central Hospital, Xiangtan, China

**Keywords:** Triglyceride-glucose index, Cumulative exposure, Major adverse cardiovascular events, Insulin resistance, Type 2 diabetes

## Abstract

**Background:**

The triglyceride-glucose (TyG) index is a reliable surrogate marker of insulin resistance and is associated with major adverse cardiovascular events (MACEs) in patients with type 2 diabetes mellitus (T2DM). However, the long-term effect of the TyG index on the incidence of MACEs remains unclear. We aimed to investigate the association between the cumulative TyG index and the risk of MACEs in patients with T2DM.

**Methods:**

This post-hoc analysis of the Action to Control Cardiovascular Risk in Diabetes (ACCORD) trial assessed patients’ (T2DM > 3 months) cumulative TyG index and MACE data from the study database. Five fasting blood glucose and triglyceride measurements, at baseline and the first four visits, were taken from 5695 participants who had not experienced MACEs. Cumulative exposure to the TyG index was calculated as the weighted sum of the mean TyG index value for each time interval (value × time). Multivariable-adjusted Cox proportional hazard models and restricted cubic spline analysis were used to determine the association between the cumulative TyG index and MACEs. The incremental predictive value of the cumulative TyG index was further assessed.

**Results:**

Over a median follow-up of 5.09 years, 673 (11.82%) MACEs occurred, including 256 (4.50%) cardiovascular disease (CVD) deaths, 288 (5.06%) non-fatal myocardial infarctions (MIs), and 197 (3.46%) strokes. The risk of developing MACEs increased with the cumulative TyG index quartile. After adjusting for multiple potential confounders, the hazard ratios for the very high cumulative TyG index group versus the low group were 1.59 (95% confidence interval [CI], 1.17–2.16), 1.97 (95% CI 1.19–3.26), and 1.66 (95% CI 1.02–2.70) for overall MACEs, CVD death, and non-fatal MI, respectively. Restricted cubic spline analysis also showed a cumulative increase in the risk of MACEs with an increase in the magnitude of the cumulative TyG index. The addition of the cumulative TyG index to a conventional risk model for MACEs improved the C-statistics, net reclassification improvement value, and integrated discrimination improvement value.

**Conclusions:**

In patients with T2DM, the cumulative TyG index independently predicts the incidence of MACEs, and monitoring the long-term TyG index may assist with optimized-for-risk stratification and outcome prediction for MACEs.

*Trial registration* URL: http://www.clinicaltrials.gov. Unique identifier: NCT00000620.

**Supplementary Information:**

The online version contains supplementary material available at 10.1186/s12933-022-01599-1.

## Background

Cardiovascular disease (CVD), including coronary heart disease, cerebrovascular disease, and peripheral arterial disease, is the leading cause of morbidity and mortality in individuals with diabetes [1]. Recent cardiovascular outcome event trials in type 2 diabetes mellitus (T2DM) patients showed that the rate of major adverse cardiovascular events (MACEs), including myocardial infarction (MI), stroke, and cardiovascular death, ranges from 9.4 to 12.1% [2–4] and results in an estimated $37.3 billion in cardiovascular-related spending per year associated with diabetes [5]. Thus, assessing and controlling individual cardiovascular risk factors to prevent or slow CVD in people with diabetes is important. Insulin resistance (IR), underpinning the pathophysiology of T2DM, is commonly associated with hypertension, dyslipidemia, and dysglycemia [6]. Additionally, inflammatory and thrombotic markers are associated with IR thereby creating an inflammatory atherothrombotic syndrome [7]. Therefore, long-term IR not only causes hyperglycemia and hypertension, but it also results in abnormal blood lipid metabolism and anticoagulant mechanisms, which increases the risk of thrombosis. Furthermore, several studies have demonstrated that coronary atherosclerotic disease in diabetes is associated with more atheroma and increased macrophage infiltration and intraplaque thrombi [8]; this indicates that IR in diabetes is a biomarker of an inflammatory atherothrombotic process linking MACEs to the underlying mechanism in T2DM. Therefore, long-lasting monitoring and control of IR may contribute to the prevention of MACEs in patients with T2DM.

In clinical settings or large population-based studies, IR measurements can be challenging owing to some limitations in the current gold-standard methods (the euglycemic clamp and homeostasis model assessment for insulin resistance [HOMA-IR]) [9, 10]. The triglyceride-glucose (TyG) index, as a parameter derived from fasting blood glucose (FBG) and triglyceride (TG) levels first proposed in 2008, has been proposed as a convincing indicator of IR [11, 12]. The TyG index has been proven to correlate with the euglycemic-hyperinsulinemic clamp formation and thus has a validity similar to HOMA-IR [11]. Moreover, the TyG index has the advantage of being easily accessible in any clinical setting, thereby making our findings widely applicable. Thus, the TyG index was used as a biomarker of IR in this post-hoc analysis to study its relationship with the risk of MACEs in patients with T2DM. Recently, a considerable number of studies have provided robust statistical evidence suggesting that the TyG index may help identify individuals at risk of developing T2DM [13, 14] and that it is associated with the development and prognosis of CVD [15]. A cohort study (6078 participants > 60 years old) suggested that participants in the highest TyG index quartile were at a 72% higher risk of CVD events [16]. Using data from the National Health Information Database with a median follow-up period of 8.2 years, Hong et al. further confirmed that participants in the highest TyG index quartile were at a higher risk of stroke and MI, independently of other traditional cardiovascular risk factors [17]. The relationship between the TyG index and different types of CVD has been consecutively revealed thereafter [18]. However, most observational studies on the association between a high TyG index and cardiovascular outcomes have focused on TyG index values measured at a single timepoint, and few studies have characterized the long-term TyG index and its implications for MACEs in patients with T2DM [19–21].

More importantly, poorly controlled lipid and glucose levels influence the slow progression of cardiovascular events. Long-term versus contemporary lipid and glucose levels may more significantly impact the development of cardiovascular events [22]. Moreover, cumulative exposure in cardiovascular risk factors has been shown to predict the risk of adverse long-term cardiovascular outcomes and mortality, independent of baseline risk factors levels over [19]. Previous study showed that the longer duration of higher TyG index exposure was significantly associated with increased CVD risk [23]. Although our previous study has also shown that high long-term trajectories of the TyG index were significantly associated with the occurrence of MACEs [24], it remains unclear whether the cumulative TyG index is a stronger risk factor for future MACEs in patients with T2DM. In the present study, we hypothesized that long-term exposure to a high TyG index during follow-up may influence the development of MACEs in T2DM. Using data from the Action to Control Cardiovascular Risk in Diabetes (ACCORD) [25] and ACCORD Follow-On (ACCORDION) studies [26], we evaluated the association between cumulative TyG index exposure during early follow-up and the incidence of MACEs later in life.

## Methods

### Study population

This post-hoc analysis of a prospective study used data from the ACCORD/ACCORDION trial (ClinicalTrials.gov number, NCT00000620). The rationale and design of the ACCORD trial have been described previously [25]. Participants in the ACCORD trial were recruited between June 2001 and October 2005 from 77 sites across the United States and Canada. The trial enrolled 10,251 participants (mean age, 62 years) who had T2DM for a median duration of 10 years, a mean glycated hemoglobin (HbA1c) level of 8.3%, and previous CVD or CVD risk factors. All surviving ACCORD participants from participating sites who could be contacted were subsequently offered the opportunity to take part in the ACCORDION study, during which data on cardiovascular and other health-related outcomes and measurements were collected and analyzed between May 2011 and October 2014. The ACCORD/ACCORDION study design was approved by Wake Forest University, USA (coordinating center) and the institutional review boards at each center (participating clinical sites). Written informed consent was obtained from all participants.

### Data collection and outcomes

According to the ACCORD protocol, screened lipid levels were either measured at a local laboratory or obtained from the medical records. If obtained from medical records, the most recent values recorded within the previous 12 months were used. If there were no lipid values in the medical records within the previous 12 months, the protocol specified that a blood test must be performed by the local laboratory. The baseline lipid measurement was defined as visit_0_. The clinical follow-up timepoints were 1, 2, 3, 4, 5, 6, 7, and 8 years after the initial visit, and the blood lipid measurements were acco_6_rdingly defined as visit_1_, visit_2_, visit_3_, visit_4_, visit_5_, visit, visit_7_, and visit_8_. As previously described [27], the cumulative TyG index was developed from the baseline to the fourth visit during the first 4 years of follow-up to predict the risk of MACEs in later follow-up timepoints. Among the total study population of 10,251 participants, 4229 participants had missing data on FBG or TG at baseline or the first four visits, and 327 participants experienced MACEs during the first four visits. Ultimately, a total of 5695 participants were enrolled in the present study (Additional file 1).

The primary outcome of the study was the occurrence of MACEs after the fourth visit, including non-fatal MI, non-fatal stroke, and CVD-related death.

### Calculation of cumulative TyG index

The TyG index was calculated as: ln (fasting TG [mg/dL] × FBG [mg/dL]/2) [11]. As previously described [19, 21, 27, 28], the cumulative TyG index was defined as the summation of the average TyG index for each pair of consecutive examinations multiplied by the time between these two consecutive visits, in years:

([TyG index_visit 0_ + TyG index_visit 1_]/2 × time_0−1_) + ([TyG index_visit 1_ + TyG index_visit 2_]/2 × time_1−2_) + ([TyG index_visit 2_ + TyG index_visit 3_]/2 × time_2−3_) + ([TyG index_visit 3_ + TyG index_visit 4_]/2 × time_3−4_).

TyG index_visit 0_, TyG index_visit 1_, TyG index_visit 2_, TyG index_visit 3_, and TyG index_visit 4_ indicate the TyG index at baseline and the first, second, third, and fourth examinations, respectively, and time_0–1_, time_1–2_, time_2–3,_ and time_3–4_ indicate the participant-specific time intervals between consecutive visits in years. The mean of time_0–1,_ time_1–2_, time_2–3_, and time_3–4_ was 1 year. According to previous studies [23], the participants were stratified according to cumulative TyG index quartile: low (< 34.86, reference group), moderate (34.86–36.47), high (36.47–38.15), and very high (> 38.15).

### Statistical analysis

Baseline characteristics are presented as the mean ± standard deviation (SD) or frequency with percentage, as appropriate. Differences in the characteristics across cumulative TyG index groups were tested using analysis of variance (ANOVA) or the Kruskal–Wallis test for continuous variables according to distribution and the Chi-square test for categorical variables. The Kaplan–Meier method was used to evaluate the incidence rate of MACEs and their subtypes, and the differences between groups were evaluated using a log-rank test.

The proportional hazard assumption was evaluated by the visualization of Schoenfeld residuals, and no potential violation was observed. A Cox proportional hazard regression model was applied to calculate the hazard ratios (HRs) and 95% confidence intervals (CIs) for MACEs and their subtypes. Univariate analyses were conducted to evaluate relationships between all variables and MACEs before the multivariate Cox regression analyses. Variables with *P* < 0.20 in the univariate analyses were included in the multivariable analyses. Additionally, variables which were clinically closely related to MACEs were included in the multivariable analyses (even if *P* > 0.20 in univariable analyses) to avoid missing important conventional cardiovascular risk factors (e.g., age, sex, smoking, etc.). Three multivariable models with progressive degrees of adjustment were used to adjust for potential confounders of incident MACEs and components of MACEs. Model 1 was adjusted for age, sex, education level, race, smoking status, drinking status, years of hypertension diagnosis, years of diabetes diagnosis, depression, body mass index (BMI), systolic blood pressure, diastolic blood pressure, heart rate, and history of CVD; Model 2 was adjusted for model 1 covariables plus plasma total cholesterol, low-density lipoprotein cholesterol (LDL-C), HbA1c, and estimated glomerular filtration rate (eGFR); Model 3 was adjusted for model 2 covariables plus statin, insulin, non-dihydropyridine calcium channel blockers, dihydropyridine calcium channel blockers, thiazolidinediones, and thiazide diuretics treatment. *P*-values for trends were computed using the cumulative TyG index quartile as the ordinal variable. To capture the dose–response relationship between the cumulative TyG index and risk of MACEs, restricted cubic spline analysis was performed, with four knots at the 25th, 50th, 75th, and 95th percentiles of the cumulative TyG index distribution. The reference point for the cumulative TyG index was the median value of the reference group, and the HR was adjusted for the variables in Model 3.

The competing risk model was applied to assess the associations between the cumulative TyG index and outcomes, with non-CVD-related death being regarding as a competing risk event. Subgroup analyses were conducted on participants after stratification by age (< 65 or ≥ 65 years), sex, and BMI (< 30 or ≥ 30 kg/m^2^) to identify any modification caused by these variables. Interactions between subgroups were assessed using likelihood ratio tests, in which models with and without multiplicative interaction terms were compared. Additionally, we used C-statistics, a net reclassification index (NRI), and an integrated discrimination improvement (IDI) to evaluate the incremental predictive value of the cumulative TyG index beyond conventional risk factors.

All analyses were conducted using SPSS 23.0 (IBM, Armonk, NY, USA) and Stata 15.1 (Stata Corp LLC, TX, USA). Statistical significance was defined as a two-sided *P*-value < 0.05.

## Results

### Baseline characteristics of the participants

A total of 5695 eligible participants were included (mean age, 62.59 ± 6.50 years; 61.93% male). The baseline characteristics of participants according to cumulative TyG index quartile are presented in Table 1. The mean age differed slightly between groups. Individuals in the very high cumulative TyG index quartile (Table 1) were more likely to be male, white, smoke, have experienced previous cardiovascular events or depression, have a longer duration of hyperlipidemia or a shorter duration of diabetes, have a higher BMI, diastolic blood pressure, heart rate, HbA1c, alanine transaminase (ALT), eGFR, TC, TG, and FBG levels, and have low HDL-C. Insulin and statin use was lower in individuals in the very high cumulative TyG index quartile, whereas metformin use was higher in this group.

**Table 1:** Subject baseline characteristics by quartile of cumulative TyG index

	All N = 5695	Low n = 1424	Moderate n = 1424	High n = 1424	Very high n = 1423	*P* value
Age, y (mean ± SD)	62.59 ± 6.50	63.37 ± 6.44	63.35 ± 6.55	62.31 ± 6.64	61.35 ± 6.13	**< 0.001**
Sex, No. (%)						**0.021**
Female	2168 (38.07)	578 (40.59)	542 (38.06)	550 (38.62)	498 (35.00)	
Male	3527 (61.93)	846 (59.41)	882 (61.94)	874 (61.38)	925 (65.00)	
Education, No. (%)						
Less than high school	763 (13.40)	231 (16.22)	220 (15.45)	171 (12.01)	141 (9.91)	**< 0.001**
High school graduate or GED	1520 (26.69)	377 (26.47)	379 (26.62)	371 (26.05)	393 (27.62)	0.811
Some college	1872 (32.87)	421 (29.56)	454 (31.88)	493 (34.62)	504 (35.42)	**0.003**
College degree or higher	1537 (26.99)	394 (27.67)	370 (25.98)	388 (27.25)	385 (27.06)	0.773
Race, No. (%)						**< 0.001**
White	3644 (63.99)	667 (46.84)	893 (62.71)	1003 (79.44)	1081 (75.97)	
Non-white	2051 (36.01)	757 (53.16)	531 (37.29)	421 (29.56)	342 (24.03)	
Living alone, No. (%)	1110 (19.49)	295 (20.72)	274 (19.24)	264 (18.54)	277 (19.47)	0.525
Depression, No. (%)	1258 (22.09)	224 (15.73)	291 (20.44)	364 (25.56)	379 (26.63)	**< 0.001**
Previous cardiovascular event, No. (%)	1892 (33.22)	431 (30.27)	466 (32.72)	485 (34.06)	510 (35.84)	**0.014**
Duration of hypertension, y (mean ± SD)	10.20 ± 9.61	10.18 ± 9.70	10.07 ± 9.54	10.25 ± 9.55	10.28 ± 9.68	0.959
Duration of diabetes, y (mean ± SD)	10.65 ± 7.40	11.64 ± 7.76	11.25 ± 7.61	10.48 ± 7.42	9.24 ± 6.54	**< 0.001**
Duration of hyperlipemia, y (mean ± SD)	5.78 ± 5.57	5.16 ± 5.14	5.50 ± 5.50	5.69 ± 5.41	6.68 ± 6.05	**< 0.001**
Cigarette-smoking, No. (%)						**< 0.001**
Yes	3280 (57.59)	748 (52.53)	791 (55.55)	851 (59.76)	890 (62.54)	
No	2415 (42.41)	676 (47.47)	633 (44.45)	573 (40.24)	533 (37.46)	
Alcohol, No. (%)						0.785
Yes	1419 (24.92)	349 (24.51)	356 (25.00)	368 (24.84)	346 (24.31)	
No	4274 (75.05)	1075 (75.49)	1066 (74.86)	1056 (74.16)	1077 (75.69)	
BMI, kg/m^2^ (mean ± SD)	32.21 ± 5.32	31.26 ± 5.51	32.02 ± 5.32	32.60 ± 5.25	32.97 ± 5.03	**< 0.001**
Blood pressure, mmHg (mean ± SD)						
Systolic	136.35 ± 16.66	136.40 ± 16.62	136.42 ± 17.04	136.53 ± 16.27	136.04 ± 16.71	0.876
Diastolic	75.12 ± 10.45	74.25 ± 10.29	74.24 ± 10.31	75.74 ± 10.65	76.24 ± 10.40	**< 0.001**
Heart rate, bpm (mean ± SD)	72.56 ± 11.68	71.69 ± 11.81	72.16 ± 11.48	72.95 ± 11.85	73.43 ± 11.50	**< 0.001**
Medications, No. (%)						
Insulin	1942 (34.10)	526 (34.94)	535 (37.57)	467 (32.79)	414 (29.09)	**< 0.001**
Metformin	3694 (64.86)	886 (62.22)	897 (62.99)	949 (66.64)	962 (67.60)	**0.004**
ACEI/ARB	3946 (69.29)	982 (68.96)	992 (69.66)	970 (68.12)	1002 (70.41)	0.587
Aspirin	3131 (54.98)	790 (55.48)	821 (57.65)	759 (53.30)	761 (53.48)	0.066
Statin	3638 (63.88)	919 (64.54)	958 (67.28)	882 (61.94)	879 (61.77)	**0.006**
Cholesterol absorption inhibitors	86 (1.51)	11 (0.77)	22 (1.54)	29 (2.04)	24 (1.69)	**0.043**
Non-dihydropyridine calcium channel blockers	464 (8.15)	125 (8.78)	123 (8.64)	105 (7.37)	111 (7.80)	0.454
Dihydropyridine calcium channel blockers	649 (11.40)	188 (13.20)	151 (10.60)	160 (11.24)	150 (10.54)	0.089
Thiazolidinediones	1302 (22.86)	331 (23.24)	342 (24.02)	313 (21.98)	316 (22.21)	0.538
Thiazide diuretics	1575 (27.66)	423 (29.71)	383 (26.90)	400 (28.09)	369 (25.93)	0.135
Glycated hemoglobin, % (mean ± SD)	8.25 ± 1.01	8.20 ± 0.96	8.22 ± 1.00	8.25 ± 1.04	8.32 ± 1.04	**0.006**
ALT, mg/dL (mean ± SD)	27.71 ± 15.13	25.29 ± 12.96	26.41 ± 14.12	28.63 ± 15.77	30.51 ± 16.86	**< 0.001**
eGFR, mL/min/1.73 m^2^ (mean ± SD)	91.26 ± 27.39	92.03 ± 23.92	89.73 ± 22.99	90.16 ± 24.11	93.11 ± 36.25	**0.002**
Total plasma cholesterol, mg/dL (mean ± SD)	183.23 ± 41.24	173.61 ± 36.10	177.49 ± 36.75	184.78 ± 38.70	197.06 ± 48.32	**< 0.001**
Plasma LDL-C, mg/dL (mean ± SD)	104.81 ± 32.94	104.57 ± 30.90	104.64 ± 31.14	106.54 ± 33.55	103.50 ± 35.89	0.100
Plasma HDL, mg/dL (mean ± SD)	41.59 ± 11.31	47.32 ± 12.74	42.75 ± 10.62	40.00 ± 9.79	36.30 ± 8.77	**< 0.001**
Fasting plasma glucose, mg/dL (mean ± SD)	175.38 ± 55.21	157.22 ± 50.45	170.93 ± 52.41	180.61 ± 54.71	192.76 ± 56.90	**< 0.001**
Plasma triglycerides, mg/dL (mean ± SD)	190.88 ± 154.79	108.60 ± 55.14	151.09 ± 68.68	192.96 ± 85.59	310.97 ± 240.93	**< 0.001**
Cumulative TyG	36.56 ± 2.45	33.54 ± 1.05	35.71 ± 0.45	37.28 ± 0.48	39.72 ± 1.43	**< 0.001**
Incident MACEs, No. (%)	673 (11.82)	132 (9.27)	169 (11.87)	171 (12.01)	201 (14.13)	**0.001**
CVD death, No. (%)	256 (4.50)	51 (3.58)	70 (4.92)	56 (3.93)	79 (5.55)	**0.044**
Non-fatal MI	288 (5.06)	46 (3.23)	73 (5.13)	75 (5.27)	94 (6.61)	**0.001**
Non-fatal stroke	197 (3.46)	47 (3.30)	43 (3.02)	59 (4.14)	48 (3.37)	0.398

Data are presented as mean ± SD or as n (%)

The participants were stratified by cumulative TyG index quartile: low (< 34.86, reference group), moderate (34.86–36.47), high (36.47–38.15), and very high (> 38.15)

The bold values indicate that comparison between groups with significant statistical significance (*P* value < 0.05)

*ACEI/ARB* angiotensin-converting enzyme inhibitors/angiotensin receptor blockers, *ALT* alanine transaminase, *BMI* body mass index, *BPM* beats per minute, *CVD* cardiovascular disease, *eGFR* estimated glomerular filtration rate, *GED* general education development, *HDL-C* high-density lipoprotein cholesterol, *LDL-C* low-density lipoprotein cholesterol, *MACEs* major adverse cardiovascular events, *MI* myocardial infarction, *SD* standard deviation, *TyG* triglyceride-glucose

P-value for the test of the difference across quartiles of cumulative TyG index using the chi-square test (categorical variables), analysis of variance (continuous variables), or Kruskal–Wallis test (nonparametric comparisons)

### Association of cumulative TyG index with MACEs

**Fig. 1:**
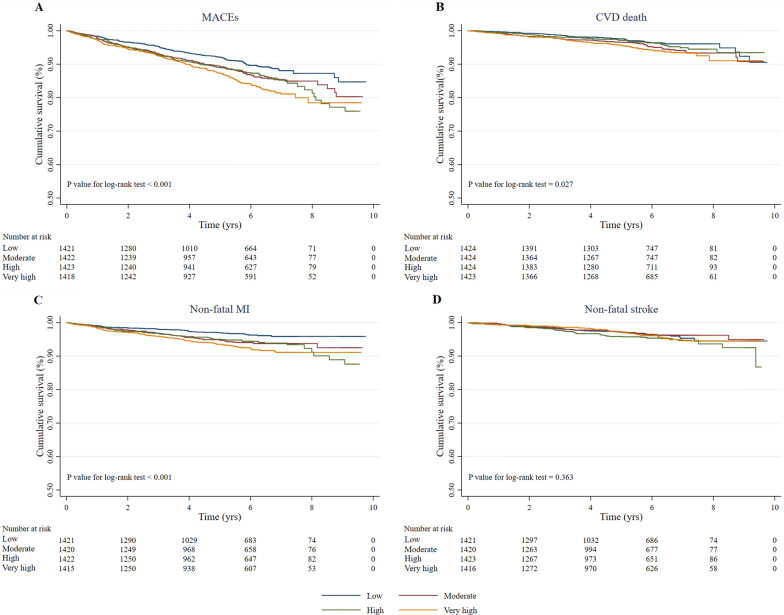
Kaplan–Meier survival curves for MACEs and individual outcomes based on cumulative TyG index quartiles. **a** MACEs; **b** cardiovascular disease (CVD)-related death; **c** non-fatal myocardial infarction (MI); **d** non-fatal stroke. *MACEs* major adverse cardiovascular events, *TyG* triglyceride-glucose

Over a median follow-up of 5.09 years, 673 (11.82%) MACEs were identified, including 256 (4.50%) CVD-related deaths, 288 (5.06%) non-fatal MIs, and 197 (3.46%) strokes. The incidence of MACEs increased substantially with the magnitude of cumulative TyG index (quartiles), reaching a maximum incidence of 14.13% in the very high quartile group (Table 1). Similarly, the risks of CVD-related death and non-fatal MI also increased with increasing quartiles of cumulative TyG index. Kaplan–Meier curves were used to determine the probability of MACEs and individual outcomes (Fig. 1). The probability of poor patient outcomes (MACEs, CVD-related death, and non-fatal MI) was significantly higher in patients with a high cumulative TyG index than in those with a low cumulative TyG index (*P* < 0.05).

**Fig. 2:**
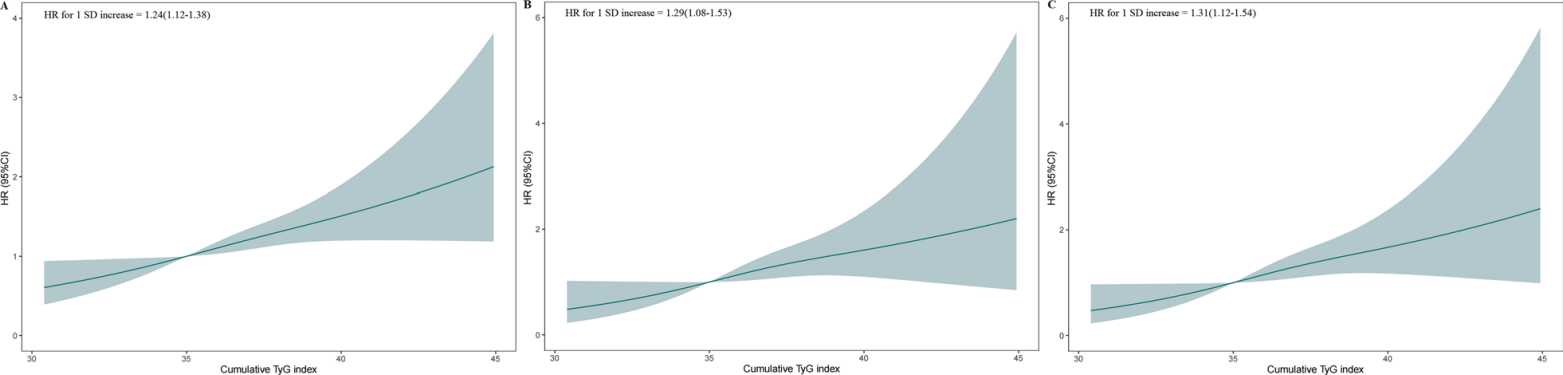
Multivariable-adjusted hazard ratios for MACEs and individual outcomes based on restricted cubic spline analysis. Restricted cubic spline analysis has five knots at the 25th, 50th, 75th, and 95th percentiles of changes in the triglyceride-glucose (TyG) index. **a** MACEs; **b** cardiovascular disease (CVD)-related death; **c** non-fatal myocardial infarction (MI). *CI* confidence interval, *CVD* cardiovascular disease, *HR* hazard ratio, *MACEs* major adverse cardiovascular events, *SD* standard deviation, *TyG* triglyceride-glucose

The cumulative risk of MACEs also increased according to the magnitude of the cumulative TyG index, and this trend remained significant even after adjusting for potential confounding factors in Model 3 (*P* for trend < 0.001; Table 2). The HRs were 1.59 (95% CI 1.17–2.16), 1.38 (95% CI 1.03–1.86), and 1.24 (95% CI 0.94–1.64) for the very high, high, and moderate groups, respectively, compared to the low cumulative TyG index group after adjusting for potential confounding factors (Table 2). In Model 3, when measuring the cumulative TyG index as a continuous variable, a 1-SD increase in cumulative TyG index was associated with a 24% higher risk of MACEs after fully adjusting for all potential confounders (HR, 1.24; 95% CI 1.12–1.38; *P* < 0.001; Fig. 2A). These findings suggest that the cumulative TyG index could be used as a predictor of MACEs. Furthermore, when evaluating associations between the cumulative TyG index and individual outcomes, we found graded associations of the cumulative TyG index with CVD-related death and non-fatal MI (*P* for trends = 0.028 and < 0.001, respectively) but not non-fatal stroke. After adjusting for potential confounders, significant differences in CVD-related death and non-fatal MI were still observed between the very high and low groups. Additionally, when measuring the cumulative TyG index as a continuous variable, a 1-SD increase in the cumulative TyG index was associated with higher risks of CVD-related death and non-fatal MI (Fig. 2B, C, Table 2). These findings indicate that the cumulative TyG index correlated with MACEs and could be used as a prognostic marker for patients with T2DM.

**Table 2:** Risk of incident MACEs and individual outcomes for the cumulative TyG index

Cumulative TyG index	Events/No. at risk	Unadjusted		Model 1		Model 2		Model 3	
		HR (95% CI)	*P* value	HR (95% CI)	*P* value	HR (95% CI)	*P* value	HR (95% CI)	*P* value
MACEs									
Low	132/1292	Ref.		Ref.		Ref.		Ref.	
Moderate	169/1255	1.32 (1.05–1.66)	**0.016**	1.26 (0.96–1.66)	0.095	1.25 (0.94–1.65)	0.122	1.24 (0.94–1.64)	0.136
High	171/1253	1.34 (1.07–1.69)	**0.011**	1.39 (1.05–1.83)	**0.019**	1.37 (1.02–1.83)	**0.035**	1.38 (1.03–1.86)	**0.030**
Very high	201/1,222	1.63 (1.31–2.03)	**< 0.001**	1.62 (1.23–2.15)	**0.001**	1.58 (1.16–2.13)	**0.003**	1.59 (1.17–2.16)	**0.003**
P for trend			**< 0.001**		**< 0.001**		**< 0.001**		**< 0.001**
CVD death									
Low	51/1424	Ref.		Ref.		Ref.		Ref.	
Moderate	70/1424	1.40 (0.97–2.01)	0.069	1.40 (0.89–2.20)	0.142	1.32 (0.84–2.09)	0.235	1.32 (0.84–2.08)	0.235
High	56/1424	1.11 (0.76–1.63)	0.577	1.37 (0.86–2.18)	0.183	1.34 (0.82–2.17)	0.240	1.31 (0.80–2.13)	0.281
Very high	79/1423	1.63 (1.14–2.31)	**0.007**	2.05 (1.29–3.26)	**0.002**	1.92 (1.16–3.16)	**0.011**	1.97 (1.19–3.26)	**0.008**
P for trend			**0.028**		**0.004**		**0.016**		**0.017**
Non-fatal MI									
Low	46/1424	Ref.		Ref.		Ref.		Ref.	
Moderate	73/1424	1.64 (1.14–2.37)	**0.008**	1.59 (1.03–2.46)	**0.037**	1.58 (1.01–2.46)	**0.044**	1.64 (1.05–2.57)	**0.030**
High	75/1424	1.68 (1.16–2.42)	**0.006**	1.64 (1.05–2.57)	**0.030**	1.55 (0.97–2.49)	0.066	1.61 (1.00–2.59)	**0.049**
Very high	94/1423	2.17 (1.52–3.09)	**< 0.001**	1.69 (1.08–2.63)	**0.021**	1.66 (1.03–2.68)	**0.039**	1.66 (1.02–2.70)	**0.041**
P for trend			**< 0.001**		**0.002**		**0.005**		**0.004**
Non-fatal stroke									
Low	47/1424	Ref.		Ref.		Ref.		Ref.	
Moderate	43/1424	0.94 (0.62–1.41)	0.751	0.98 (0.60–1.60)	0.930	1.01 (0.61–1.65)	0.979	0.97 (0.58–1.60)	0.891
High	59/1424	1.29 (0.88–1.90)	0.187	1.30 (0.81–2.07)	0.277	1.30 (0.80–2.12)	0.290	1.29 (0.79–2.12)	0.312
Very high	48/1423	1.08 (0.72–1.61)	0.707	1.23 (0.75–2.02)	0.409	1.26 (0.73–2.17)	0.416	1.29 (0.75–2.23)	0.363
P for trend			0.381		0.134		0.133		0.115

Model 1: Adjusted for age, sex, education level, race, smoking status, drinking status, years of hypertension diagnosis, years of diabetes diagnosis, depression, body mass index, systolic blood pressure, diastolic blood pressure, heart rate, and history of CVD

Model 2: Adjusted for model 1 covariables plus plasma total cholesterol, HbA1c, LDL-C, and eGFR

Model 3: Adjusted for model 2 covariables plus statin, insulin, non-dihydropyridine calcium channel blockers, dihydropyridine calcium channel blockers, thiazolidinediones, and thiazide diuretics treatment

The bold values indicate that comparison between groups with significant statistical significance (*P* value < 0.05)

*CI* confidence interval, *CVD* cardiovascular disease, *HR* hazard ratio, *MACEs* major adverse cardiac events, *MI* myocardial infarction, *TyG* index triglyceride-glucose index

### Subgroup analyses

**Fig. 3:**
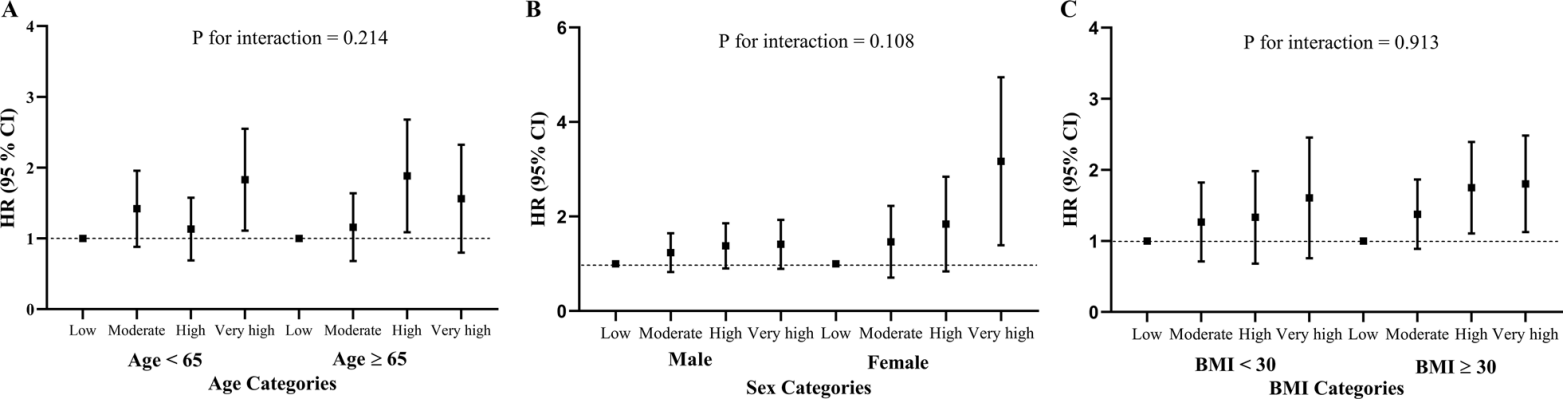
Subgroup analyses of the relationship between cumulative triglyceride-glucose (TyG) index and MACEs. The study population was stratified by age (< 65 years vs. ≥ 65 years), sex (male vs. female), and body mass index (BMI; < 30 kg/m^2^ vs. ≥ 30 kg/m^2^). Adjustments were made for age, sex, education level, race, smoking status, drinking status, years of hypertension diagnosis, years of diabetes diagnosis, depression, body mass index, systolic blood pressure, diastolic blood pressure, heart rate, history of CVD, plasma total cholesterol, HbA1c, LDL-C, eGFR, statin, insulin, non-dihydropyridine calcium channel blockers, dihydropyridine calcium channel blockers, thiazolidinediones, and thiazide diuretics treatment at the baseline level. *BMI* body mass index, *CI* confidence interval, *CVD* cardiovascular disease, *eGFR* estimated glomerular filtration rate, *HbA1c* glycated hemoglobin, *HR* hazards ratio, *LDL-C* low-density lipoprotein cholesterol, *MACEs* major adverse cardiovascular events, *SD* standard deviation, *TyG* triglyceride-glucose

To explore the associations between the cumulative TyG index and MACEs in greater detail, we performed subgroup analyses stratified by age, sex, and BMI (Fig. 3). The associations of the cumulative TyG index with the risk of MACEs and individual outcomes were consistent across subgroups. There were no significant interactions between the cumulative TyG index and age (< 65 years vs. ≥ 65 years), sex (male vs. female), or BMI (< 30 kg/m^2^ vs. ≥ 30 kg/m^2^) (*P* for interaction > 0.05 for all). Sensitivity analyses with the competing risk model (Additional file 2) also indicated good robustness of the results.

### Incremental predictive value of the cumulative TyG index

To assess the predictive value of the cumulative TyG index, we further analyzed the C-statistics, NRI, and IDI (Table 3). The C-statistics of the conventional model significantly improved with the addition of the cumulative TyG index (from 0.6864 to 0.6937, *P* = 0.032). Additionally, the risk reclassification and discriminatory power also appeared to be substantially better, with an IDI of 0.5% (95% CI 0.1–1.10; *P* < 0.001), and a NRI of 6.90% (95% CI 0.8–12.30; *P* = 0.02). Similar results were observed when CVD death and non-fatal MI. These findings indicated that adding the cumulative TyG index improved the prediction efficiency for MACEs.

**Table 3:** Incremental predictive value of the cumulative TyG index

	C statistics		NRI		IDI	
	Estimate (95% CI)	*P*	Estimate (95% CI), %	*P*	Estimate (95% CI), %	*P*
MACEs						
Conventional model	0.6864 (0.66–0.71)		Ref.		Ref.	
Conventional model + cumulative TyG index	0.6937 (0.67–0.72)	**0.032**	6.90 (0.80–12.30)	**0.02**	0.50 (0.1–1.10)	**< 0.001**
CVD death						
Conventional model	0.7247 (0.69–0.76)		Ref.		Ref.	
Conventional model + cumulative TyG index	0.7292 (0.69–0.77)	0.368	4.10 (− 2.4–13.20)	**0.09**	4.00 (0.00–1.70)	**< 0.001**
Non-fatal MI						
Conventional model	0.6808 (0.64–0.72)		Ref.		Ref.	
Conventional model + cumulative TyG index	0.6895 (0.65–0.73)	0.118	12.60 (0.70–19.70)	**0.03**	0.40 (0.00–1.20)	**0.02**
Non-fatal stroke						
Conventional model	0.6704 (0.62–0.72)		Ref.		Ref.	
Conventional model + cumulative TyG index	0.6723 (0.63–0.72)	0.5421	5.00 (− 6.10 to 14.50)	0.289	0.00 (− 0.10 to 0.60)	0.507

The conventional model was adjusted for age, sex, education level, race, smoking status, drinking status, years of hypertension diagnosis, years of diabetes diagnosis, depression, body mass index, systolic blood pressure, diastolic blood pressure, heart rate, history of CVD, plasma total cholesterol, HbA1c, LDL-C, eGFR, statin, insulin, non-dihydropyridine calcium channel blockers, dihydropyridine calcium channel blockers, thiazolidinediones, and thiazide diuretics treatment at the baseline level

The bold values indicate that comparison between groups with significant statistical significance (*P* value < 0.05)

*CI* confidence interval, *CVD* cardiovascular disease, *IDI* integrated discrimination improvement, *MACEs* major adverse cardiovascular events, *MI* myocardial infarction, *NRI* net reclassification index, *TyG* index triglyceride-glucose index

## Discussion

In the present post-hoc analysis of the ACCORD cohort, we found that the cumulative TyG index was significantly associated with the risk of MACEs. Notably, the risk of MACEs and TyG index over time were directly proportional. Similar patterns were observed for CVD-related death and non-fatal MI. These trends remained when subjected to sensitivity analyses and analyses of stratified data. Furthermore, the addition of the cumulative TyG index to the baseline risk model, including traditional risk factors, significantly improved its predictive value.

The comparisons conducted on baseline characteristics across the quartiles in present study found that participants with a very high cumulative TyG index were associated with high cardiovascular risk when comprised with those with a low cumulative TyG index. However, diabetes duration is longer in the lower cumulative TyG index group indicating patients with longer diabetes duration would be likely to better control of blood glucose and lipid levels. Notably, the causality relationship needs to be further clarified by prospective study. Regarding cardiovascular events, the incidence of MACEs increased substantially with the magnitude of cumulative TyG index, which is consistent with previous studies [29]. Nevertheless, cumulative TyG index is not significantly associated with non-fatal stroke when analyzing the relationship between cumulative index and individual events. Despite previous studies showing that high cumulative TyG index is associated with a higher risk of ischemic stroke by using data from the Kailuan cohort [21], we found that cumulative TyG was not related to non-fatal stroke in patients with T2DM. One major reason for this difference could be the inclusion criteria of participants; previous studies have focused primarily on general population, while we concentrated on participants who had T2DM.

Previous observational studies on the association between the TyG index and cardiovascular outcomes focused on the TyG index at a single timepoint and ignored changes in the TyG index over time, which could result in potential regression dilution bias and subsequently affect the accuracy of results. Recently, the association between cumulative exposure in various biological measures (e.g., blood pressure and cholesterol) and incident CVD have been examined [30, 31]. Cui et al. described the cumulative effect of TyG on the risk of cardiovascular disease [23]. The risk of developing CVD increased with the cumulative TyG index, after adjustment for multiple potential confounders. Moreover, this study proposed that the causal effect of the cumulative TyG index on the risk of CVD is determined by both the magnitude and the cumulative duration of exposure to a high TyG index. While cardiovascular damage evolves over prolonged periods, current prediction models use only a ‘snapshot’ of baseline data as a proxy for actual long-term exposure to risk factors. Furthermore, within person variability and measurement errors bias risk prediction may occur if based on a single measurement. This suggests a need to consider both the magnitude and the cumulative duration of exposure to risk factors. Therefore, evaluation of the TyG index at baseline alone does not reflect the longitudinal association between the cumulative exposure to high TyG index and cardiovascular outcomes over time. It is feasible that the participant's electronic medical record allows the rapid integration of data across multiple time points, allowing the calculation of a cumulative exposure index. Using the cumulative TyG index in the present study, we found that a higher cumulative TyG index resulted in an increased risk of developing MACEs in T2DM patients. The risk of MACE development was highest in individuals in the highest quartile group, with a multivariate-adjusted HR of 1.72 (95% CI 1.28–2.30). In addition, this risk was not attenuated after adjusting for traditional risk factors and clinical variables. The cumulative effect seems to be independent of the pathogenesis of MACEs. Likewise, the longest exposure time was associated with the highest risk of MACEs. Nowadays, handheld and mobile technology essentially place a continuous monitoring device in the entire population’s pocket, and many health systems record all information electronically [32], making a compelling case for using routinely collected electronic health information to furthers assess the utility of repeated and cumulative measurements of risk factors.

The current study extends the findings of these previous reports and the cumulative TyG index to the conventional risk model, which had an incremental effect on the predictive value for incidence of MACEs. The predictive utility of a single TyG index value for the prediction of MACEs has shown that the TyG index is a useful parameter and a stronger predictive factor than HbA1c and TG levels for cardiovascular events; therefore, it may offer an additional prognostic benefit in patients with T2DM [33]. Recently, an observational study that included 1932 patients with acute coronary syndrome (ACS) and T2DM further confirmed that the TyG index was an independent predictor of MACEs in patients with T2DM and ACS (OR, 2.32; 95% CI 1.92–2.80) [34]. Additionally, a study that enrolled 776 patients with ACS and T2DM collected percutaneous coronary intervention evidence that the TyG index was also an independent predictor of MACEs (HR, 2.17; 95% CI 1.45–3.24) [35]. Importantly, a study showed that the addition of the TyG index to the traditional Framingham predictors improved the predictive accuracy of coronary heart disease by using data from the Vascular Metabolic CUN (Clinica Universidad de Navarra) cohort [15]. By using data from the Kailuan study with a median follow-up period of 7.01 years, Wang et al. further confirmed that substantial changes in the TyG index independently predicted the risk of CVD in the general population [20]. In the present study, the findings further imply that the cumulative TyG index is associated with a high risk of incidence of MACEs and that this higher risk can be predicted more accurately with the addition of the cumulative TyG index than with models that only included a single TyG index value. This highlights the importance of monitoring longitudinal patterns of the cumulative TyG index in clinical practice for patients with T2DM.

The pathophysiological processes of atherosclerosis suggest a mechanistic association between MACEs and the cumulative TyG index. TyG is an index consisting of two risk factors for CVD: lipid- and glucose-related factors, which are reflective of IR in the human body. Numerous studies have confirmed the TyG index as a reliable marker of IR, which may explain this association [12]. IR can induce glucose metabolism imbalance, which contributes to hyperglycemia and in turn induces inflammation, oxidative stress, and excessive glycosylation; this ultimately promotes smooth muscle cell proliferation, collagen crosslinking, and collagen deposition. These pathological events contribute to increased vascular stiffness and aging [7]. Additionally, systemic lipid disturbances induced by IR may cause the initiation and progression of atherosclerosis [36]. Furthermore, IR mitigates nitric oxide bioavailability and promotes oxidative stress in endothelial cells [37–39], and it increases adhesion-induced and thromboxane A2-dependent tissue factor expression in platelets [40], which have been implicated in the occurrence of both thrombosis and cardiovascular events. Thus, an increase in the TyG index over time may accelerate the development of arterial stiffness, thereby leading to the development of MACEs in patients with T2DM.

These findings have substantial clinical implications. IR, a well-established hallmark of metabolic disorders and systemic inflammation, is not only a substantial risk factor for CVD but also contributes to a worse prognosis. Current studies have confirmed that the TyG index can be used as a reliable and convenient surrogate for IR, and it may be more optimized for risk stratification and outcome prediction for MACEs in patients with T2DM. Additionally, a recent systematic review found moderate-to-low quality evidence about the usefulness of the TyG index as a surrogate biochemical marker of IR [41]. Due to the lack of a standardized IR definition and heterogeneity between studies, further validation and standardized cut-off values are needed in clinical practice. Clinical practice is currently guided by contemporary FBG and TG values, whereas our findings suggest that incorporating cumulative exposure to IR into clinical practice may further refine risk assessment of cardiovascular outcomes and help inform strategies for secondary prevention of MACEs in patients with T2DM. Additionally, the TG/HDL-C ratio was considered a potential predictive marker of IR and β-cell dysfunction [42]. It is closely associated with T2DM as well as CVD development. The possible mechanisms could be referenced to that high TG levels reflect the abundance of TG-rich lipoproteins particles containing apo B and in essence, highly atherogenic, that it may help to explain the pathophysiological processes of atherosclerosis. Moreover, given that the risk of developing MACEs is associated with the cumulative TyG index, it is plausible that improving IR and maintaining optimal FBG and TG levels may prevent the development of MACEs better than the current paradigm of deferring IR improvement to later, when MACEs have likely to already have occurred.

The strengths of this study include its large cohort size and its complete follow-up of MACEs and repeat assessment of FBG and TG measurements, which allowed us to model the long-term cumulative TyG index and assess associations between the cumulative TyG index and MACEs. These advantages not only permitted us to reliably estimate the cumulative TyG index beyond conventional risk in patients with T2DM with high cardiovascular risk, but they also demonstrated that patients with T2DM with a high cumulative TyG index had a greater risk of MACEs. This suggests that this population may benefit from earlier and more frequent screening for adverse cardiovascular events, aggressive risk factor management, and maintenance of metabolic health.

This study had some limitations. First, our findings were observational, and the causal role of the TyG index on cardiovascular event risk should be verified in further prospective intervention studies. Second, despite adjustments for potential known confounding variables in the multivariable Cox regression analysis, we cannot exclude a possible residual bias because of the post-hoc nature of this study, nor can we assess all metabolic factors and parameters related to IR. Finally, the population of the ACCORD trial included only high-risk patients with T2DM. Additional studies are necessary to increase the generalizability of the results.

## Conclusions

In T2DM, long exposure to a high TyG index may increase the risk of MACEs. These findings indicate that monitoring the longitudinal TyG index may assist with optimized-for-risk stratification, as well as outcome prediction for MACEs in patients with T2DM.

## Supplementary Information

**Additional file 1.** Flowchart for study participant selection from the Action to Control Cardiovascular Risk in Diabetes (ACCORD) study. FBG, fasting blood glucose; *MACEs* major adverse cardiovascular events, *TG* triglyceride, *TyG* triglyceride-glucose.

**Additional file 2.** Sensitivity analyses for the association of cumulative triglyceride-glucose (TyG) index from visit 0 to visit 4 with MACEs and individual outcomes. *CI* confidence interval, *CVD* cardiovascular disease, *HR* hazards ratio, *MACEs* major adverse cardiovascular events, *MI* myocardial infarction.

## Data Availability

The datasets used and analyzed during the current study are available from the ACCORD/ACCORDION Research Materials obtained from the National Heart, Lung, and Blood Institute (NHLBI) Biologic Specimen and Data Repository Information Coordinating Center, https://biolincc.nhlbi.nih.gov/studies/accord/.

## Abbreviations

ACCORD: Action to Control Cardiovascular Risk in Diabetes
ACEI/ARB: Angiotensin-converting enzyme inhibitors/angiotensin receptor blockers
ACS: Acute coronary syndrome
BMI: Body mass index
CI: Confidence interval
CVD: Cardiovascular disease
eGFR: Estimated glomerular filtration rate
FBG: Fasting blood glucose
GED: General education development
HbA1c: Glycated hemoglobin
HDL-C: High-density lipoprotein cholesterol
HOMA-IR: Homeostasis model assessment for insulin resistance
HR: Hazards ratio
IR: Insulin resistance
LDL-C: Low-density lipoprotein cholesterol
MACE: Major adverse cardiovascular event
MI: Myocardial infarction
OR: Odds ratio
ROC: Receiver operating characteristic
SD: Standard deviation
T2DM: Type 2 diabetes mellitus
TG: Triglyceride
TyG: Triglyceride-glucose
