# Supplementary figures and images for: Association of the cumulative triglyceride-glucose index with major adverse cardiovascular events in patients with type 2 diabetes
Source: Cardiovasc Diabetol. 2022 Aug 23;21:161. doi: 10.1186/s12933-022-01599-1 (PMC9400318; doi:10.1186/s12933-022-01599-1)

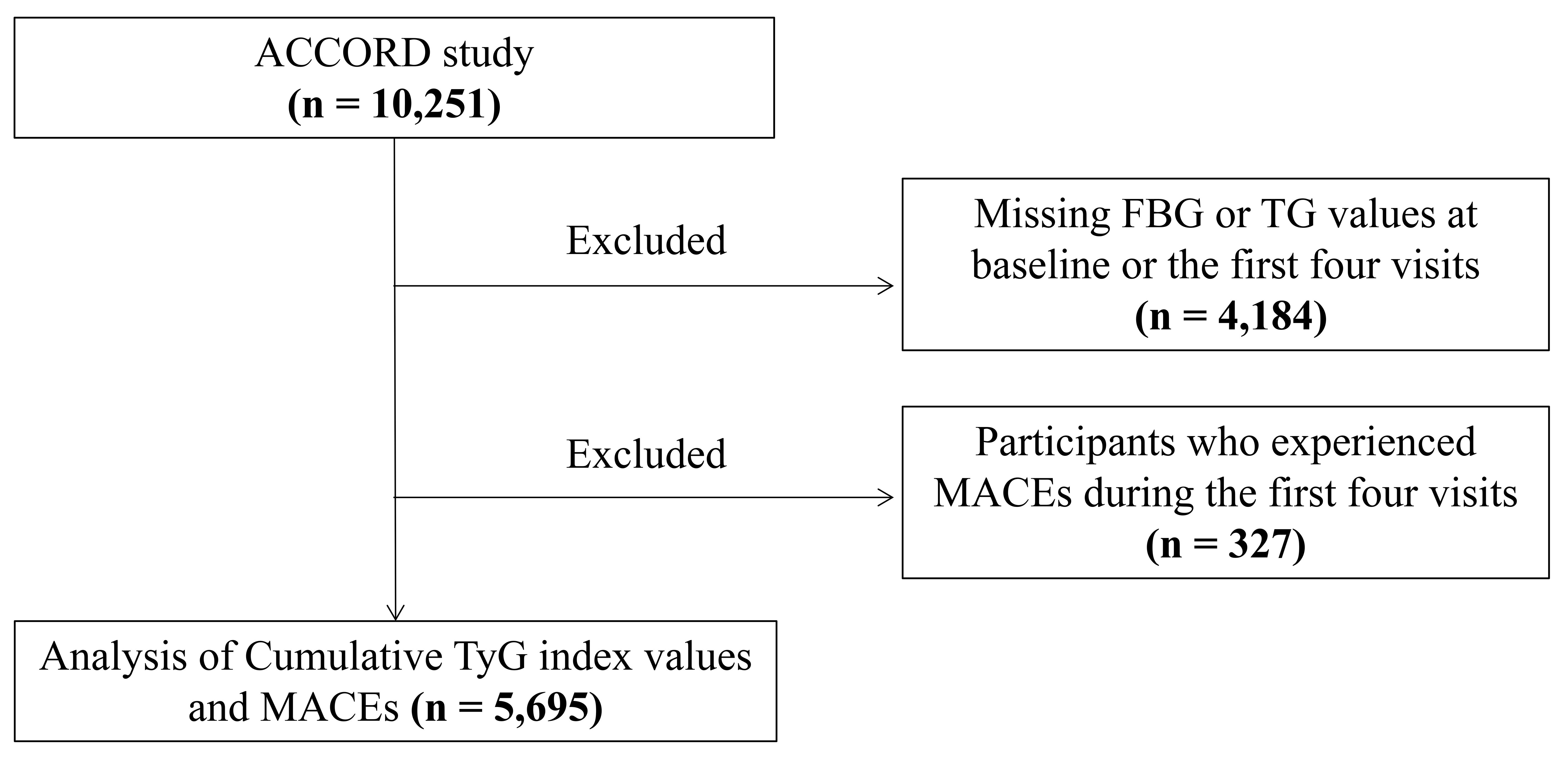

Supplement: Supplementary file 1 — Additional file 1. Flowchart for study participant selection from the Action to Control Cardiovascular Risk in Diabetes (ACCORD) study. FBG, fasting blood glucose; MACEs major adverse cardiovascular events, TG triglyceride, TyG triglyceride-glucose. [file 12933_2022_1599_MOESM1_ESM.tif]

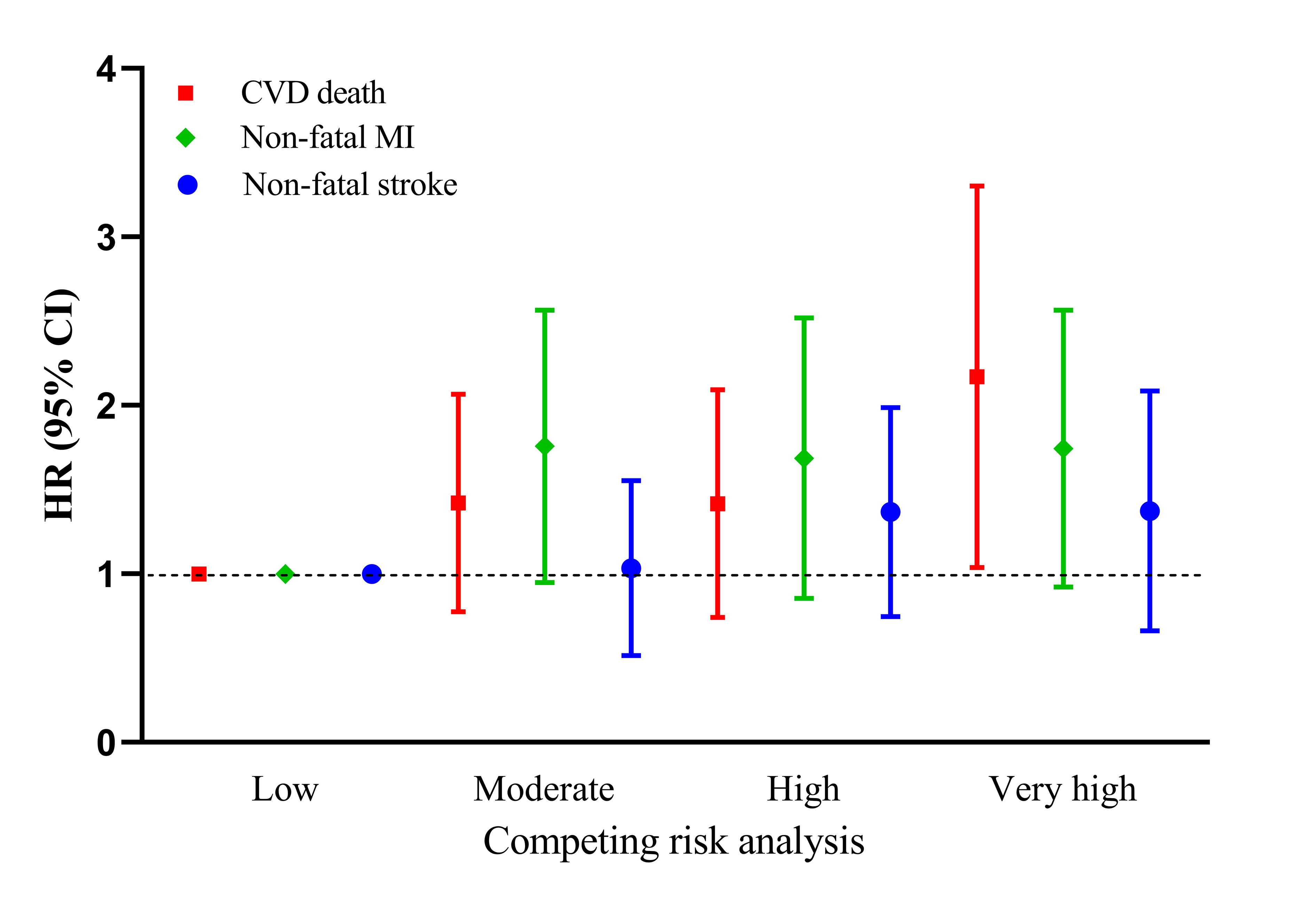

Supplement: Supplementary file 2 — Additional file 2. Sensitivity analyses for the association of cumulative triglyceride-glucose (TyG) index from visit 0 to visit 4 with MACEs and individual outcomes. CI confidence interval, CVD cardiovascular disease, HR hazards ratio, MACEs major adverse cardiovascular events, MI myocardial infarction. [file 12933_2022_1599_MOESM2_ESM.tif]
